# Non-Invasive Monitoring of Cerebral Edema Using Ultrasonic Echo Signal Features and Machine Learning

**DOI:** 10.3390/brainsci14121175

**Published:** 2024-11-23

**Authors:** Shuang Yang, Yuanbo Yang, Yufeng Zhou

**Affiliations:** 1 State Key Laboratory of Ultrasound in Medicine and Engineering, Chongqing Medical University, Chongqing 400016, China; yshuang0619@163.com (S.Y.); yuanboy107@gmail.com (Y.Y.); 2 Chongqing Key Laboratory of Biomedical Engineering, Chongqing Medical University, Chongqing 400016, China; 3 National Medical Products Administration (NMPA), Key Laboratory for Quality Evaluation, Ultrasonic Surgical Equipment, 507 Gaoxin Ave., Donghu New Technology Development Zone, Wuhan 430075, China

**Keywords:** cerebral edema, ultrasonic echo signal, non-invasive monitoring, machine learning

## Abstract

Objectives: Cerebral edema, a prevalent consequence of brain injury, is associated with significant mortality and disability. Timely diagnosis and monitoring are crucial for patient prognosis. There is a pressing clinical demand for a real-time, non-invasive cerebral edema monitoring method. Ultrasound methods are prime candidates for such investigations due to their non-invasive nature. Methods: Acute cerebral edema was introduced in rats by permanently occluding the left middle cerebral artery (MCA). Ultrasonic echo signals were collected at nine time points over a 24 h period to extract features from both the time and frequency domains. Concurrently, histomorphological changes were examined. We utilized support vector machine (SVM), logistic regression (LogR), decision tree (DT), and random forest (RF) algorithms for classifying cerebral edema types, and SVM, RF, linear regression (LR), and feedforward neural network (FNNs) for predicting the cerebral infarction volume ratio. Results: The integration of 16 ultrasonic features associated with cerebral edema development with the RF model enabled effective classification of cerebral edema types, with a high accuracy rate of 97.9%. Additionally, it provided an accurate prediction of the cerebral infarction volume ratio, with an *R*^2^ value of 0.8814. Conclusions: Our proposed strategy classifies cerebral edema and predicts the cerebral infarction volume ratio with satisfactory precision. The fusion of ultrasound echo features with machine learning presents a promising non-invasive approach for the monitoring of cerebral edema.

## 1. Introduction

Cerebral edema is a pathological state characterized by an increase in brain volume due to an abnormal increase or accumulation of intracranial fluid [1]. Cerebral edema is often a complication of traumatic brain injury, stroke, and other craniocerebral diseases, and cerebral edema can lead to elevated intracranial pressure (ICP), causing neurological deficits, brain displacement, or herniation [2]. In extreme cases, it poses a serious threat to life. The etiology of cerebral edema is intricate, with two primary types: cytotoxic and vasogenic cerebral edema [3]. Cytotoxic cerebral edema typically predominates initially, while vasogenic cerebral edema takes over later [4]. This distinction is crucial, as it influences treatment strategies [5,6]. Therefore, timely classification and ongoing monitoring of cerebral edema are essential for improving diagnostic precision and therapeutic efficiency.

Ultrasound methods are excellent candidates for investigating cerebral edema due to their non-invasive nature. During cerebral edema, brain tissue properties like density and hardness change, affecting the acoustic impedance of the intracranial brain tissue and ultrasound propagation inside it. It is hypothesized that ultrasound echo features are correlated with the types and severity of cerebral edema. Machine learning, particularly pattern recognition and classification algorithms, may assist in classifying cerebral edema types and predicting its severity based on features extracted from ultrasonic echo signals, potentially improving diagnostic precision and real-time monitoring capabilities. In this study, a non-invasive method of monitoring cerebral edema was introduced using ultrasonic echo signal features and machine learning, aiming to validate the feasibility of this approach.

To meet the study’s objectives, a series of experiments were conducted. A cerebral edema model was established by permanently occluding the left MCA in rats. The ultrasonic echo signals were then recorded at nine time points within 24 h to extract the 22 features from waveform and spectra. The morphology of cerebral edema was quantitatively analyzed. Finally, selected features were input into machine learning models to classify cerebral edema types and predict cerebral infarction volume ratios, with a comparison of the performance of different models.

## 2. Literature Review

### 2.1. Research Related to Cerebral Edema Monitoring

Currently, the methods for monitoring cerebral edema are classified into invasive and non-invasive types. Invasive ICP monitoring, which involves the insertion of pressure sensors into the brain parenchyma, ventricles, or subdural space, only indirectly indicating the severity of cerebral edema [7]. However, the probe is prone to shifting during monitoring, diminishing the monitoring accuracy, and carries the risk of intracranial hemorrhage or infection [8,9,10]. Lietke et al. correlated computed tomography (CT)-based cerebral edema classification with ICP (*r* = 0.51, *p* < 0.0001) [9]. Electrical impedance tomography (EIT), another invasive method, measures electrical impedance differences to assess cerebral edema progression [10], but it lacks the ability to quantify edema severity. Non-invasive imaging techniques like CT and magnetic resonance imaging (MRI) accurately delineate edema characteristics and depict its location [11]. However, their limitations, including size, cost, and ionizing radiation risks, make them unsuitable for real-time, continuous monitoring at bedside. Near-infrared spectroscopy (NIRS) has been explored for early detection of cerebral edema before ICP increases [12], and magnetic induction phase shift (MIPS) technology has shown promise in distinguishing between cytotoxic and vasogenic cerebral edema with high sensitivity and anti-interference capabilities [13]. While these studies highlight the potential for cerebral edema monitoring, advancements and practical applications are still required.

### 2.2. Research Related to Ultrasonic Echo Features and Machine Learning

Ultrasonic echo techniques, celebrated for their non-invasive, non-destructive, repeatable, real-time, and ionization-radiation-free nature, have found extensive applications in the medical field. They are utilized for monitoring or diagnosing ICP, cerebral blood flow, hydrocephalus, intracranial hematomas, and midline shift (MLS) of the brain, among other cranial conditions [14,15,16]. Transcranial color-coded Doppler (TCCD) has been shown to accurately detect intracranial hemorrhage or ischemic stroke with a sensitivity of 94% and a specificity of 95%, which aligns closely with CT imaging results [15]. Furthermore, ultrasound has also demonstrated good agreement with CT in assessing MLS in neurocritical care patients (*r* = 0.65, *p* < 0.001) [16]. The integration of machine learning with ultrasonic echo signal features has demonstrated exceptional performance in classification or prediction. By extracting features from the time domain, frequency domain after Fourier transform, and time–frequency domain after wavelet transform of the ultrasound echo signal, and then training using machine learning models for classification or prediction, a dynamic and promising research frontier has emerged [17,18,19,20,21,22,23]. For instance, a combination of six features from the ultrasonic radiofrequency (RF) spectrum with a neural network classifier achieved an accuracy (ACC) of 91%, a sensitivity of 92%, and a specificity of 90% in identifying prostate cancer [20]. The use of ultrasonic echo signal features (mean, variance, median, kurtosis, root mean square, and skewness) in blood, in conjunction with support vector machine (SVM), successfully distinguished varying degrees of red blood cell (RBC) aggregation, with an ACC of 85.88 ± 2.99% [21]. SVM and random forest (RF) classifiers, leveraging ultrasonic RF signal, B-mode sonographic texture, and attenuation features, classified benign and malignant breast diseases with an area under the curve (AUC) and receiver operating characteristic (ROC) of 0.86 and 0.81, respectively [22]. Spectrum analysis of the ultrasonic echo signal, employing the partial least squares regression method, predicted the porcine intramuscular fat content, yielding a correlation coefficient of 0.76 between predicted and actual fat values [23]. Consequently, such strategies hold significant promise in disease classification and monitoring, and using ultrasonic echo signal features in conjunction with machine learning to monitor cerebral edema may be a feasible approach.

## 3. Materials and Methods

### 3.1. Experimental Animals and Grouping

Sixty healthy adult Sprague–Dawley (SD) rats weighing 250 ± 20 g without specific pathogens or infections and evenly divided by sex were obtained from the Animal Experiment Center of Chongqing Medical University. The research protocol was approved and supervised by the Institutional Animal Care and Use Committee (IACUC) of Chongqing Medical University (IACUC-CQMU-2024-0643). The study adhered to the Guide in the Care and Use of Laboratory Animals established by the U.S. National Academy of Sciences. The rats were maintained in a temperature-controlled environment of 20–25 °C, under a 12/12 h light–dark cycle, with ad libitum access to food and water. They were randomly assigned to three groups: (1) 20 rats with cerebral edema and 3 with sham surgery controls were monitored using ultrasound echoes before and at nine postoperative time points within 24 h (0, 1, 2, 3, 6, 9, 12, 18, and 24 h); (2) 27 rats were used for 2,3,5-triphenyltetrazolium chloride (TTC) staining at each time point (3 rats per point); (3) 10 rats were utilized for histological assessment.

### 3.2. Cerebral Ischemia Model

Rats were deprived of food and water for 12 h prior to surgery. Following anesthesia induction with pentobarbital sodium (1.5%, 0.2 mL/100 g, P3761, Sigma-Aldrich, St. Louis, MO, USA), a permanent cerebral ischemia model was established via middle cerebral artery occlusion (MCAO) using a silicone thread plug (45-0480, Hengqinda, Shanghai, China) [24,25]. A 1.5–2 cm neck incision was made 1 cm away the left of the midline, and the left common carotid artery (CCA), external carotid artery (ECA), and internal carotid artery (ICA) were carefully isolated along the sternocleidomastoid muscle. The vagus nerve was meticulously separated to prevent injury that could affect respiration and swallowing. The proximal end of the CCA and the origin of the ECA were ligated with surgical thread, and a hemostatic clamp and surgical threads were positioned at the origin of the ICA and distal end of the CCA, respectively. An incision was made about 5 mm between the CCA’s end and the carotid bifurcation. A silicone thread plug was inserted in the direction of the CCA, advancing 1–2 mm into the ICA after releasing the hemostatic clamp. The blood supply to the brain was obstructed by tightening the thread with the prepared surgical thread. Finally, the incision was closed, and rats were placed on a 37 °C thermostatic heating pad for recovery.

### 3.3. Assessment of Neurological Deficits

Neurological deficits were assessed two hours post-ischemia using the Zea–Longa method [26]: 0 = no deficits; 1 = inability to extend the uninjured forelimb; 2 = walking towards the undamaged side; 3 = turning toward the undamaged side in a rear-end shape; 4 = consciousness disturbance, no independent ambulation. Scores between 1 and 3 indicate successful modeling; otherwise, the rats were discarded.

### 3.4. Analysis of Cerebral Infarction Volume

After the rats were anesthetized, their hearts were perfused with normal saline. Then, brains were quickly extracted and refrigerated at −20 °C for 25 min. The cerebellum and other parts were removed to retain the brain tissue only, and five coronal sections of a thickness of about 2 mm were prepared. These sections were incubated in a 2% solution of TTC (Solarbio, Beijing, China) at 37 °C for 30 min and then fixed in a 4% paraformaldehyde (PFA) solution (Biosharp, Hefei, China) for 5 h in the dark. The infarct volume was analyzed using ImageJ software (1.54g, National Institute of Health, Bethesda, MD, USA), with the ratio calculated as follows [27]:

(1) $$\mathrm{infarction}\;\mathrm{volume}\;\mathrm{ratio}=\left(\frac{\mathrm{volume}\;\mathrm{of}\;\mathrm{the}\;\mathrm{contralateral}\;\mathrm{hemisphere}-\mathrm{unstained}\;\mathrm{volume}\;\mathrm{of}\;\mathrm{the}\;\mathrm{ipsilateral}\;\mathrm{hemisphere}}{\mathrm{volume}\;\mathrm{of}\;\mathrm{the}\;\mathrm{contralateral}\;\mathrm{hemisphere}}\right)\times 100\%$$

### 3.5. Assessment of Histology

Following anesthesia, rats were perfused with normal saline, and brains were rapidly removed and fixed in 4% PFA for 24 h. Paraffin sections of 3–4 μm thickness were stained with hematoxylin (H9627, Sigma-Aldrich)-eosin (E4009, Sigma-Aldrich) (HE) and examined under a 200× upright microscope (Eclipse Ci-L, Nikon, Tokyo, Japan).

### 3.6. Ultrasonic Echo Signal Acquisition

**Figure 1:**
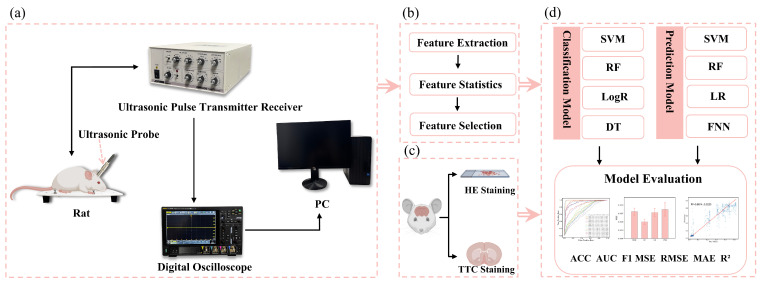
Flowchart of research framework: (**a**) acquisition of ultrasonic echoes from cerebral edema rats, (**b**) extraction, statistical analysis, and selection of ultrasonic features, (**c**) HE and TTC staining of cerebral edema rats, and (**d**) classification of cerebral edema types and prediction of cerebral infarction volume ratio.

Figure 1a shows the ultrasonic echo signal acquisition system in this study. The rats’ heads were shaved, and ultrasonic coupling gel was evenly applied. A 2.25 MHz flat ultrasound transducer (V306-SU, Olympus, Waltham, MA, USA) was used with a pulse transmitter receiver (DPR300, BYK-Gardner, New York, NY, USA) to emit and receive ultrasonic waves, which were acquired by an oscilloscope (DHO4000, RIGOL, Suzhou, China) at the sampling rate of 500 MSa/s, averaged 64 times. The ultrasound transducer was positioned over the left parietal bone, and four measurements were collected at each time point.

### 3.7. Feature Parameter Extraction

The acquired ultrasound echoes were processed to remove the DC component and then transformed into a frequency domain using Fourier analysis. Subsequently, MATLAB (R2022a, MathWorks, Natick, MA, USA) was used to extract 13 time-domain features (maximum, minimum, mean, peak-to-peak, absolute average, variance, standard deviation, kurtosis, skewness, root mean square, shape factor, impulse factor, and crest factor) and 9 frequency-domain features (mean magnitude in the frequency domain, centroid frequency, mean square frequency, variance frequency, frequency variance, mean frequency, total power, average power, and peak frequency) as listed in Table 1. These features were compiled into a dataset.

### 3.8. Classification of Cerebral Edema and Model Evaluation

Python 3.12 and PyCharm 2023.1 were employed for data analysis. The ultrasonic features dataset was constructed from 20 rats with cerebral edema at nine time points and four measurements per time point, for a total of 720 samples. The dataset was refined using the z-score method, which involved replacing outliers with the median, filling in missing values with the mean, and normalizing the data [28]. Four traditional machine learning models, including SVM, logistic regression (LogR), decision tree (DT), and RF, were used to classify cerebral edema. These classifications were each based on both a single feature and sixteen features. To fully evaluate the classification performance, a 10-fold cross validation was conducted to assess the ACC, AUC, and F1 score (F1).

### 3.9. Prediction of Cerebral Infarction Volume Ratio and Model Evaluation

Cubic spline interpolation was used to address the data imbalance between ultrasound features and cerebral infarction volume ratios by constructing a piecewise polynomial interpolation curve based on three infarction volume ratios at each time point. This method generates smooth, high-resolution interpolated results, effectively filling gaps in sparse data points. SVM, RF, linear regression (LR), and feedforward neural network (FNNs) were used to predict the volume ratio of cerebral infarction. To prevent overfitting, 10-fold cross validation was performed to determine the average values of the root mean square error (RMSE), mean square error (MSE), mean absolute error (MAE) and *R*^2^ as the evaluation indexes. The SVM model applied a linear kernel with the regularization parameter (C) of 1. RF hyperparameters included 100 trees, a maximum depth of 20, and the minimum five samples and one sample required to split an internal and leaf node, respectively. The FNN model employed the ReLU activation function with two fully connected layers (64 and 32 neurons), the Adam optimizer, the MSE loss function, a learning rate of 0.001, 50 training epochs, and a batch size of 64.

### 3.10. Statistical Analysis

Statistical analysis was conducted using Origin software (2024, OriginLab, Northampton, MA, USA). Non-parametric Kruskal–Wallis analysis and Dunn’s post-hoc test were applied to compare the features at each time point, with *p* < 0.05 indicating statistical significance.

## 4. Results

### 4.1. Changes to Features over Time

**Figure 2:**
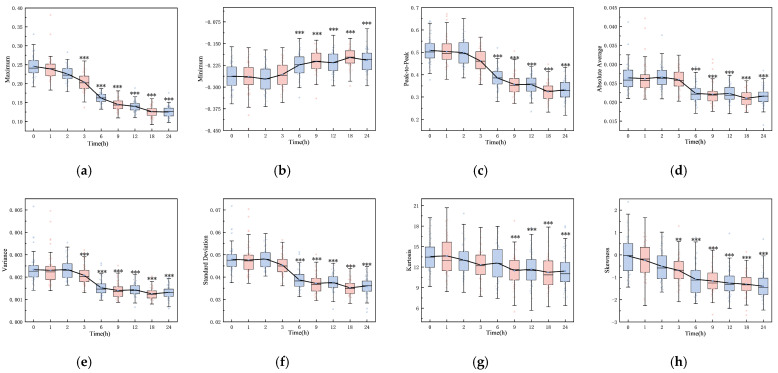

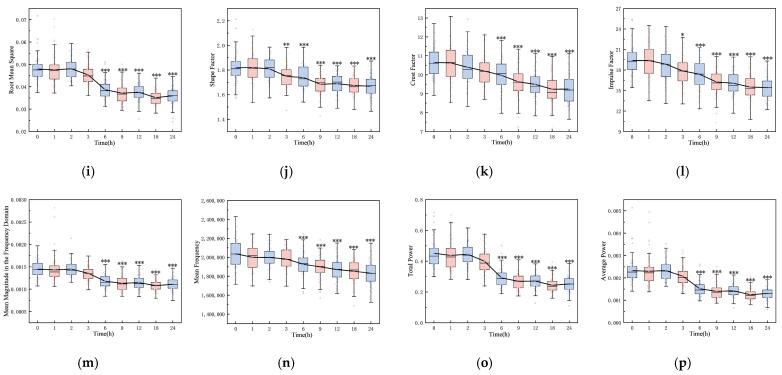
Boxplots of 16 features over time: (**a**) maximum, (**b**) minimum, (**c**) peak-to-peak, (**d**) absolute average, (**e**) variance, (**f**) standard deviation, (**g**) kurtosis, (**h**) skewness, (**i**) root mean square, (**j**) shape factor, (**k**) crest factor, (**l**) impulse factor, (**m**) mean magnitude in the frequency domain, (**n**) mean frequency, (**o**) total power, and (**p**) average power (*: *p* < 0.05, **: *p* < 0.01, ***: *p* < 0.001).

Figure 2 shows the boxplots of 16 features, highlighting significant differences between the initial (0 h) and final (24 h) time points (*p* < 0.001), including maximum, minimum, peak-to-peak, absolute average, variance, standard deviation, kurtosis, skewness, root mean square, shape factor, impulse factor, crest factor, mean magnitude in the frequency domain, mean frequency, total power, and average power. The majority of these features remained stable within the first three hours post-hemorrhagic brain injury. However, a noticeable decline was observed between 3 and 24 h post-ischemic brain injury, with the exception of a rising trend in the minimum value. Except for kurtosis at 6 h (*p* = 0.393), all features from 6 to 24 h varied significantly from the initial state (*p* < 0.001). Notably, features from the pre-modeling phase and control group did not diverge until the 6, 9, 12, 18, and 24 h marks (*p* < 0.001).

### 4.2. Volume Ratio of Cerebral Infarction over Time

**Figure 3:**
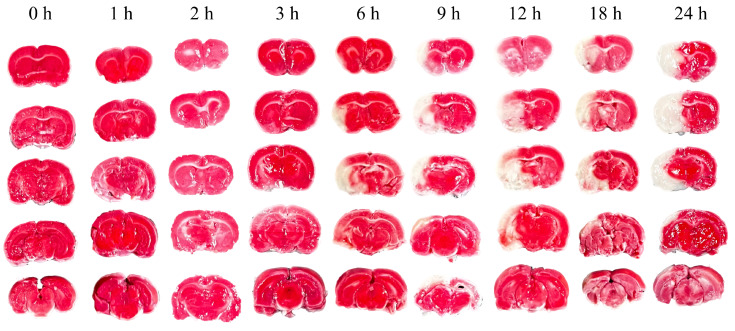
Representative TTC staining of rat brain at nine time points from 0 h to 24 h.

**Figure 4:**
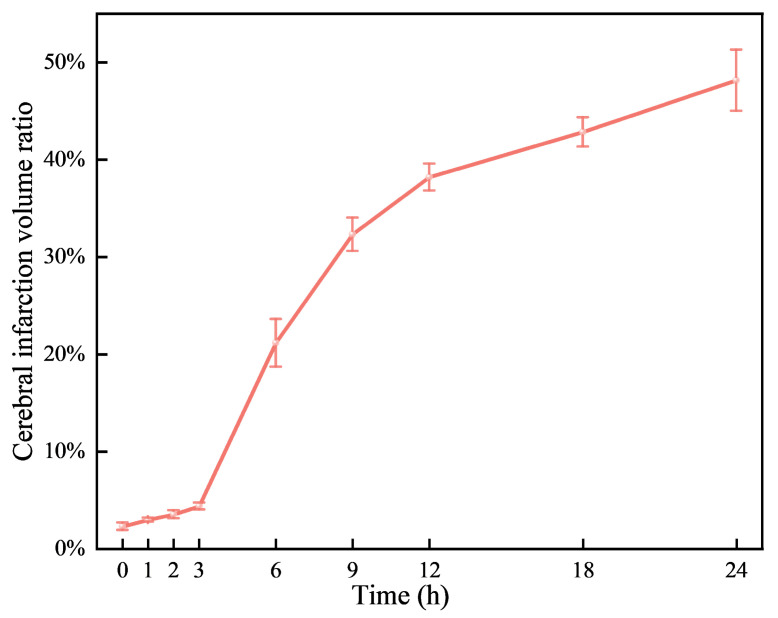
Variations of cerebral infarction volume ratio over time.

Neurological deficits were markedly evident in rats 2 h post-permanent cerebral ischemia, with a neurological score of 2.66 ± 0.48. Figure 3 illustrates representative TTC staining results at nine time points, differentiating normal (red) from ischemic (white) brain tissue. The infarcted area of ischemic brain tissue increased substantially over time. As depicted in Figure 4, the infarction volume ratio was minimally altered in the first 3 h, subsequently reaching 48.2% by the 24 h mark.

### 4.3. Pathological Morphology at Different Times

**Figure 5:**
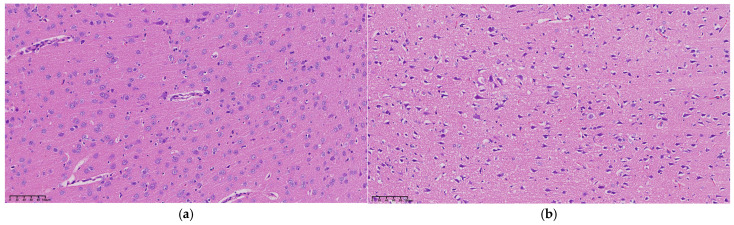

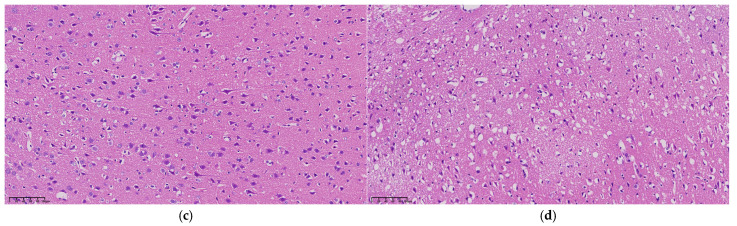
Microscopic structure of representative cerebral edema tissue at (**a**) 0 h, (**b**) 3 h, (**c**) 9 h, and (**d**) 24 h under an upright microscope at 200×, respectively.

Examination under a 200× upright microscope revealed the microstructure of brain tissue, as shown in Figure 5. At 0 h, no significant abnormalities were observed, all neurons appearing intact and neatly arranged without signs of swelling or necrosis (see Figure 5a). In contrast, 3 h post-MCAO, ischemic brain tissue showed disordered neuronal arrangement and significantly enlarged neuron volume (see Figure 5b). Nuclei appeared hyperchromatic with evident nuclear pyknosis and degeneration, indicating early cerebral edema. By 9 h, nuclear pyknosis became more pronounced, with shrunken neuronal cell bodies, enlarged intercellular spaces, and expanded perivascular spaces by fluid exudation, suggesting worsening edema and vasogenic changes (see Figure 5c). At 24 h, extensive neuronal necrosis was apparent, with edematous vacuoles, reduced cell density, and enlarged tissue gaps, indicative of severe cerebral edema (see Figure 5d).

### 4.4. Cerebral Edema Classification

**Figure 6:**
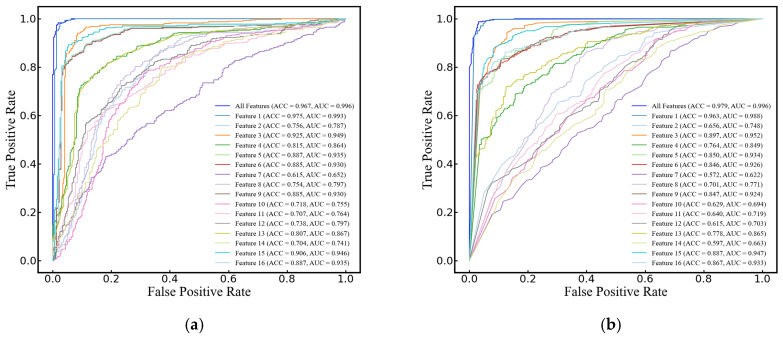

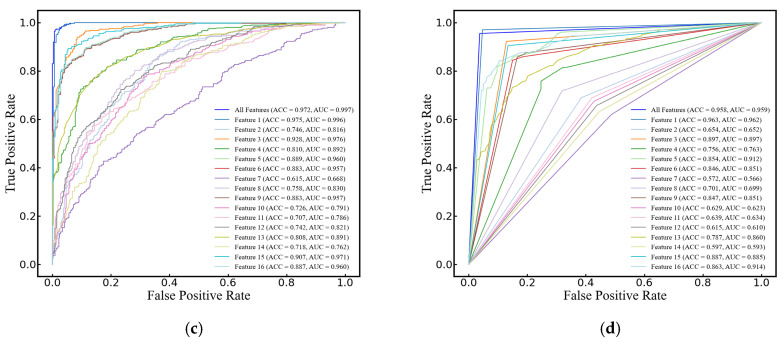
ROC curves of single and 16 features for cerebral edema classification using (**a**) SVM, (**b**) RF, (**c**) LogR, and (**d**) DT models.

In this study, SVM, RF, LogR, and DT were selected to compare their performance in classifying cerebral edema using the 16 features listed in Table 2. All models demonstrated high classification accuracy, with ACCs of 96.7 ± 3.4%, 97.9 ± 1.9%, 97.2 ± 3.0%, and 95.8 ± 2.1%, respectively. Their AUCs were 0.996 ± 0.007, 0.996 ± 0.005, 0.997 ± 0.005, and 0.959 ± 0.022, respectively, and F1 scores were 0.971 ± 0.029, 0.982 ± 0.017, 0.975 ± 0.026, and 0.962 ± 0.018, respectively. It is worth noting that when classifying with a maximum of a single feature, the yielded prediction across all four models is quite satisfactory, with ACCs of 97.5% ± 1.7%, 96.3% ± 2.2%, 97.5% ± 3.0%, and 96.3% ± 2.2%, AUCs of 0.993 ± 0.010, 0.988 ± 0.011, 0.996 ± 0.006, and 0.962 ± 0.023, and F1 scores of 0.978 ± 0.015, 0.966 ± 0.029, 0.978 ± 0.017, and 0.966 ± 0.019, respectively (see Figure 6).

### 4.5. Prediction of Cerebral Infarction Volume Ratio

**Figure 7:**
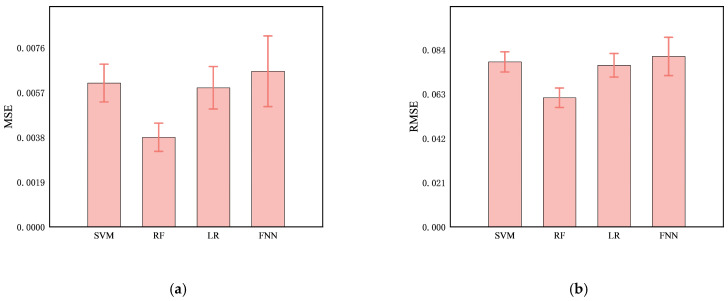

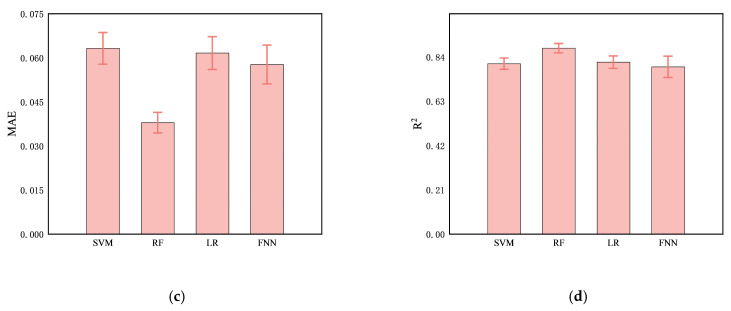
Comparison of predictive performance, (**a**) MSE, (**b**), RMSE, (**c**) MAE, and (**d**) *R*^2^, of four models (SVM, RF, LR, and FNN) for cerebral infarction volume ratio.

**Figure 8:**
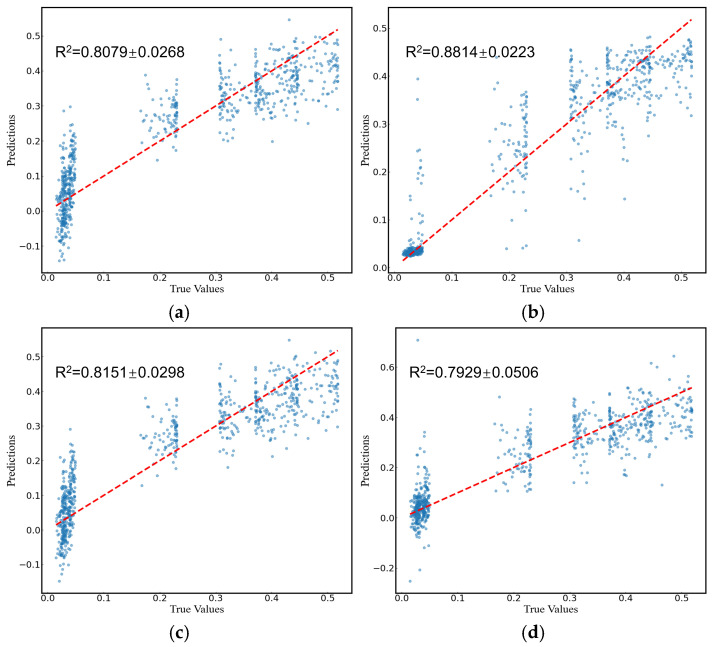
Prediction of cerebral infarction volume ratios using (**a**) SVM, (**b**) RF, (**c**) LR, and (**d**) FNN models. The dots represent the predicted or actual values, and the dashed lines represent the regression lines.

Comparing the predictive performance (MSE, RMSE, and MAE) of the four methods on the infarction volume ratio, RF outperformed the others in terms of generalization and stability, with MSE, RMSE, and MAE of 0.0038 ± 0.0006, 0.0612 ± 0.0046, and 0.0379 ± 0.0035, respectively, as shown in Table 3 and Figure 7. Additionally, the correlation between RF-predicted and actual infarction volume ratio was quite substantial (*R*² = 0.8814 ± 0.0223), as shown in Figure 8.

## 5. Discussion

Cerebral edema is frequently associated with high rates of morbidity, mortality, and disability, making its severity a critical factor in patient prognosis. Especially for patients with stroke and traumatic brain injury, real-time continuous monitoring is essential. Ultrasound technology is particularly promising for bedside and wearable monitoring. Kreft et al. rapidly detected intracranial hypertension using ultrasonic time-harmonic elastography at a shear wave speed (SWS) cutoff value of 1.67 m/s with specificity and sensitivity of 94% and 100% for the clinical ICP criteria of 25 cm H_2_O [29]. Additionally, a conformal ultrasound patch has been applied for transcranial volumetric imaging and cerebral hemodynamic monitoring [30], underscoring the vital information in the early diagnosis and monitoring of central nervous system (CNS) diseases.

In this study, features of ultrasound echo signals with machine learning were combined to enable non-invasive monitoring and classification of cerebral edema. Sixteen features of an ultrasonic echo signal related to the development of cerebral edema were extracted. It is worth noting that the wide distribution and presence of outliers in some features (see Figure 2) may be attributed to the rats’ fixation on the plate during data acquisition, a necessary step to prevent mortality from multiple anesthesia sessions within a 24 h period. However, the respiration and movement of rats may also lead to large feature variability and affect classification and prediction results.

The observed feature changes may correlate with alterations in the acoustic properties of brain tissue. Notably, the maximum value in the time domain showed a gradual decrease over time (see Figure 2a). In the early stage of cerebral edema, the influx of sodium ions, chloride ions, and water from the extracellular space into brain cells increases cell volume and tissue density, leading to higher acoustic impedance [4,6,31]. This increase enhances the reflection coefficient at the interface between edematous and normal brain tissue, resulting in stronger echoes. Pressure increases on an elastic medium are known to raise its density and ultrasound echo amplitude [32]. Interestingly, our study found a gradual decrease in the averaged maximum amplitude of echoes from the cerebral edema within the first three hours, significantly different from the 0 h mark (*p* < 0.05). This may be due to the aforementioned movement and respiration errors. With the progress of cerebral ischemia increasing, the blood–brain barrier becomes compromised, creating trans-endothelial permeability channels that allow water and certain plasma proteins to leak into the brain interstitium [4,6,31]. This leads to a decrease in brain cell volume (even cell rupture), brain tissue density, and acoustic impedance. Discrepancies in acoustic impedance are widely utilized in diagnosis [33,34,35]. High-frequency acoustic impedance imaging distinguishes between normal and cancerous breast cells with statistically different average impedance values (1.636 ± 0.010 MRayl vs. 1.612 ± 0.006 MRayl) [33]. Ito et al. also found significant acoustic impedance variations among different pathological liver conditions [34].

TTC staining at nine time points over 24 h post-cerebral edema onset revealed no distinct white ischemic brain tissue in the first three hours. Not until the sixth hour did significant white cerebral ischemic tissue occur. In comparison, another group observed obvious white ischemic brain tissue two hours after modeling [36], a difference that may be due to the type of emboli or modeling techniques used. Their cerebral infarction ratio at 24 h after successful modeling is similar to ours (49.57% vs. 48.2%), indicating a successfully established model.

Ultrasonic echo signals have emerged as a vital tool in medical research for tissue characterization, offering a more granular level of detail compared to traditional ultrasonic imaging [23]. In the study of cerebral edema, these signals revealed a significant correlation with the histological progression of the condition in rats, as evidenced by the transition from mild in the first 3 h to severe cerebral edema from 6 to 24 h. This correlation was substantiated through HE and TTC staining, which confirmed the tissue changes (see Figure 3 and Figure 4). The statistical variations in the features of ultrasonic echo signals (see Figure 2) were found to align well with the progression of cerebral edema, thereby validating their utility in predicting and classifying the condition’s advancement.

Due to different clinical treatment strategies for cytotoxic and vasogenic brain edema, distinguishing between these types is crucial for diagnosis. While the electrical resistivity of brain tissue has been utilized to differentiate edema types, the invasiveness of this technology may limit its wide application [36]. An algorithm for extracting amplitude features from electromagnetic induction has been proposed to classify acute and chronic phases of cerebral edema in rabbits, with a sensitivity of 85.0% and a specificity of 91.0% [37]. Machine learning models, including linear SVM, rough DT, and Naïve Bayes, have achieved 93% accuracy in distinguishing between cytotoxic and vasogenic brain edema in mice based on cerebral hemodynamic parameters [38]. In this study, the effectiveness of individual features was analyzed for classifying cerebral edema. It is found that not all features achieved high accuracy. For example, kurtosis alone achieved the classification accuracy of only 61.5% in the RF model. However, the synergistic utilization of multiple features improved classification accuracy, with four traditional machine learning models (SVM, LogR, DT, and RF) achieving over 95% accuracy, superior to all methods mentioned above.

In the MCAO model, the cerebral infarction volume ratio is often used to assess the severity of ischemic cerebral edema because of their positive correlation [39]. Brain water content measured using the dry-wet weight method can also demonstrate the severity of cerebral edema, but there were no significant differences between each time point [40,41]. Moreover, without precise control of time and temperature, substantial systematic errors may arise. Thus, the cerebral infarction volume ratio was utilized in this study to predict the severity of cerebral edema. The RF model showed good regression performance in predicting the cerebral infarction volume ratio, with an average MSE and *R*^2^ of 0.0038 and 0.8814, respectively. This method has great potential for non-invasive monitoring of cerebral edema in clinical settings, with either bedside or wearable designs, but requires further validation.

There are some limitations in our study. Firstly, the limited sample size may affect statistical analysis results. Secondly, the pathogenic causes and progression of cerebral edema models established using different methods may vary significantly. The performance of our non-invasive monitoring and prediction model will be evaluated using more cerebral edema models in the future. Thirdly, the rats were monitored within 24 h after the onset of cerebral edema. Long-term monitoring (e.g., 48 and 72 h) would be beneficial. Finally, more recent and advanced deep learning models, such as recurrent neural networks (RNN) and long short-term memory networks (LSTM) [42,43], will be explored to further improve accuracy.

## 6. Conclusions

This study presents a novel, non-invasive ultrasound method for the monitoring of cerebral edema. Sixteen features extracted from the ultrasound echo signals are indicative of cerebral edema progression. The integration of these features with machine learning algorithms enabled the classification of different cerebral edema types and the prediction of edema severity. The experimental outcomes are highly encouraging. The RF model exhibited superior performance in classifying cerebral edema, with an ACC of 0.979 ± 0.019, an AUC of 0.996 ± 0.005, and an F1 score of 0.982 ± 0.017. Moreover, it also effectively predicts the infarction ratio, with an *R*^2^ value of 0.8814 ± 0.0223. The findings of this research highlight the potential of ultrasound technology, in conjunction with machine learning, for assessing the severity and differentiating the types of cerebral edema. This approach offers a new, non-invasive diagnostic and real-time monitoring tool for brain trauma, which could significantly aid in clinical decision-making and patient management. Future endeavors will concentrate on expanding the dataset and exploring advanced machine learning models to enhance classification and prediction accuracy. Concurrently, efforts will be directed towards designing and fabricating wearable ultrasound transducers and a monitoring system for clinical application.

## Figures and Tables

**Table 1:** Features of ultrasonic echo signal in the time and frequency domain.

Feature	Formula	Feature	Formula
Maximum	$F_{1}=max(x)$	Impulse factor	$F_{12}=\frac{F_{4}}{F_{5}}$
Minimum	$F_{2}=min(x)$	Crest factor	$F_{13}=\frac{F_{4}}{F_{10}}$
Mean	$F_{3}=\frac{1}{N}\sum_{i=1}^{n}x_{i}$	Mean magnitude in the frequency domain	$F_{14}=\frac{1}{N}\sum_{i=1}^{n}A(f_{i})$
Peak-to-Peak	$F_{4}=F_{1}-F_{2}$	Centroid frequency	$F_{15}=\frac{\sum_{i=1}^{n}f_{i}\cdot A(f_{i})}{\sum_{i=1}^{n}A(f_{i})}$
Average	$F_{5}=\frac{1}{N}\sum_{i=1}^{n}\left|x_{i}\right|$	Mean squared frequency	$F_{16}=\frac{\sum_{i=1}^{n}{f_{i}}^{2}\cdot A(f_{i})}{\sum_{i=1}^{n}A(f_{i})}$
Variance	$F_{6}=\frac{1}{N-1}\sum_{i=1}^{n}{\left(xi-F_{3}\right)}^{2}$	Variance frequency	$F_{17}=\sqrt{F_{16}}$
Standard deviation	$F_{7}=\sqrt{F_{6}}$	Frequency variance	$F_{18}=\frac{{\sum_{i=1}^{n}(f_{i}-F_{15})}^{2}\cdot A(f_{i})}{\sum_{i=1}^{n}A(f_{i})}$
Kurtosis	$F_{8}=\frac{\frac{1}{N}\sum_{i=1}^{n}(x_{i}-F_{3}{)}^{4}}{{F_{7}}^{4}}$	Mean frequency	$F_{19}=\frac{\sum_{i=1}^{n}f_{i}\cdot P(f_{i})}{\sum_{i=1}^{n}P(f_{i})}$
Skewness	$F_{9}=\frac{\frac{1}{N}\sum_{i=1}^{n}(x_{i}-F_{3}{)}^{3}}{{F_{7}}^{3}}$	Total power	$F_{20}=\sum_{i=1}^{n}P(f_{i})$
Root mean square	$F_{10}=\sqrt{\frac{1}{N}\sum_{i=1}^{n}{x_{i}}^{2}}$	Average power	$F_{21}=\frac{1}{N}\sum_{i=1}^{n}P(f_{i})$
Shape factor	$F_{11}=\frac{F_{10}}{F_{5}}$	Peak frequency	$F_{22}=f_{arg{max}_{j}P(f_{j})}$

**Table 2:** Classification of cerebral edema using the four models.

Model	ACC	AUC	F1
SVM	0.967 ± 0.034	0.996 ± 0.007	0.971 ± 0.029
RF	0.979 ± 0.019	0.996 ± 0.005	0.982 ± 0.017
LogR	0.972 ± 0.030	0.997 ± 0.005	0.975 ± 0.026
DT	0.958 ± 0.021	0.959 ± 0.022	0.962 ± 0.018

**Table 3:** Predictive performance of four models for cerebral infarction volume ratio.

Model	MSE	RMSE	MAE	*R* ^2^
SVM	0.0061 ± 0.0008	0.0782 ± 0.0048	0.0632 ± 0.0054	0.8079 ± 0.0268
RF	0.0038 ± 0.0006	0.0612 ± 0.0046	0.0379 ± 0.0035	0.8814 ± 0.0223
LR	0.0059 ± 0.0009	0.0766 ± 0.0056	0.0616 ± 0.0056	0.8151 ± 0.0298
FNN	0.0066 ± 0.0015	0.0808 ± 0.0091	0.0577 ± 0.0066	0.7929 ± 0.0506

## Data Availability

The original contributions presented in this study are included in the article and Supplementary Materials; further inquiries can be directed to the corresponding author.

## Supplementary Materials

The following supporting information can be downloaded at: https://www.mdpi.com/article/10.3390/brainsci14121175/s1, Figures S1–S10. All raw brain sections stained with hematoxylin-eosin (HE).
