# Supplementary figures and images for: Non-Invasive Monitoring of Cerebral Edema Using Ultrasonic Echo Signal Features and Machine Learning
Source: Brain Sci. 2024 Nov 23;14(12):1175. doi: 10.3390/brainsci14121175 (PMC11674144; doi:10.3390/brainsci14121175)

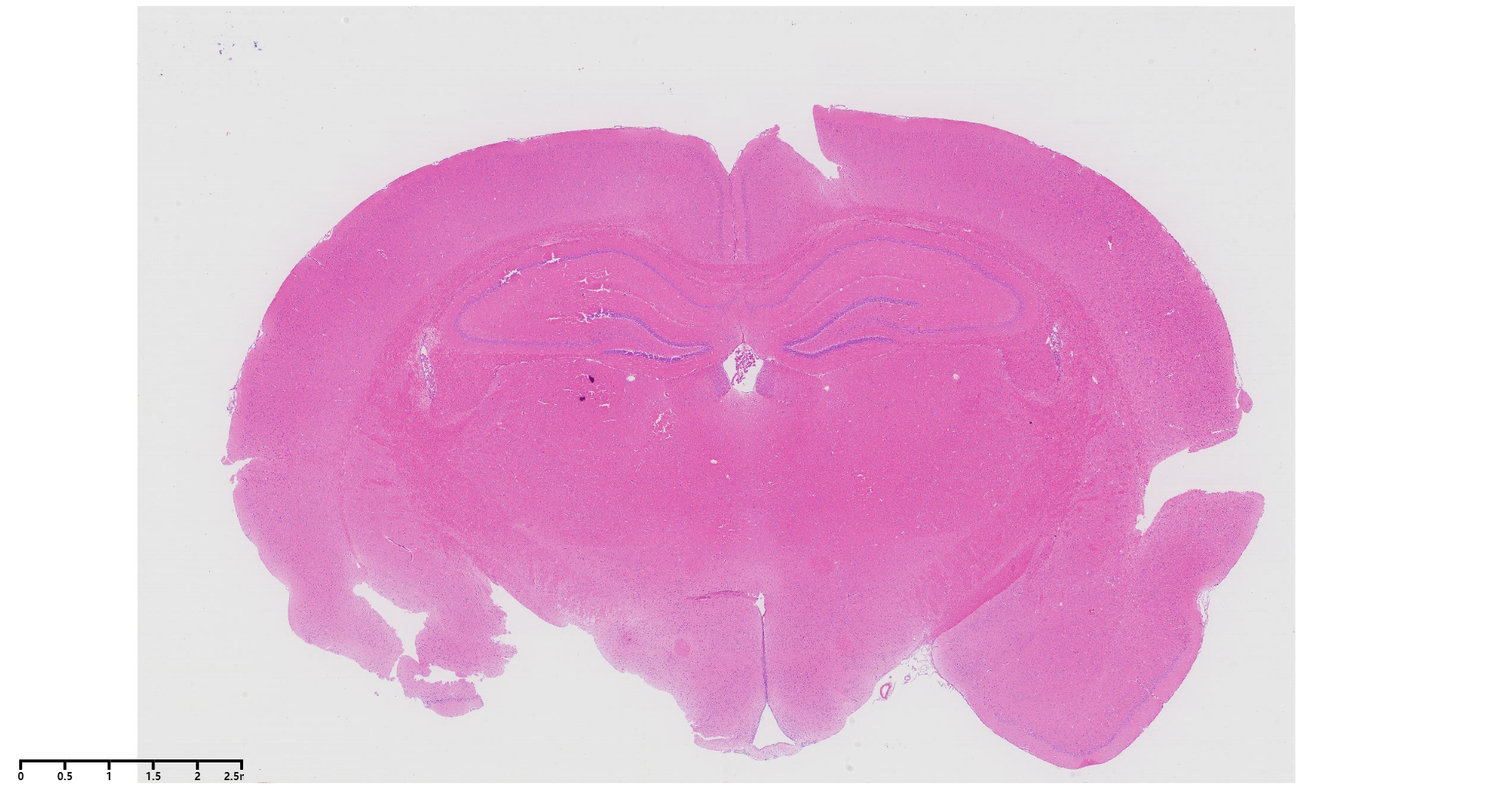

Supplement: Supplementary file 1 [file brainsci-14-01175-s001.zip › Figure 1.tif]

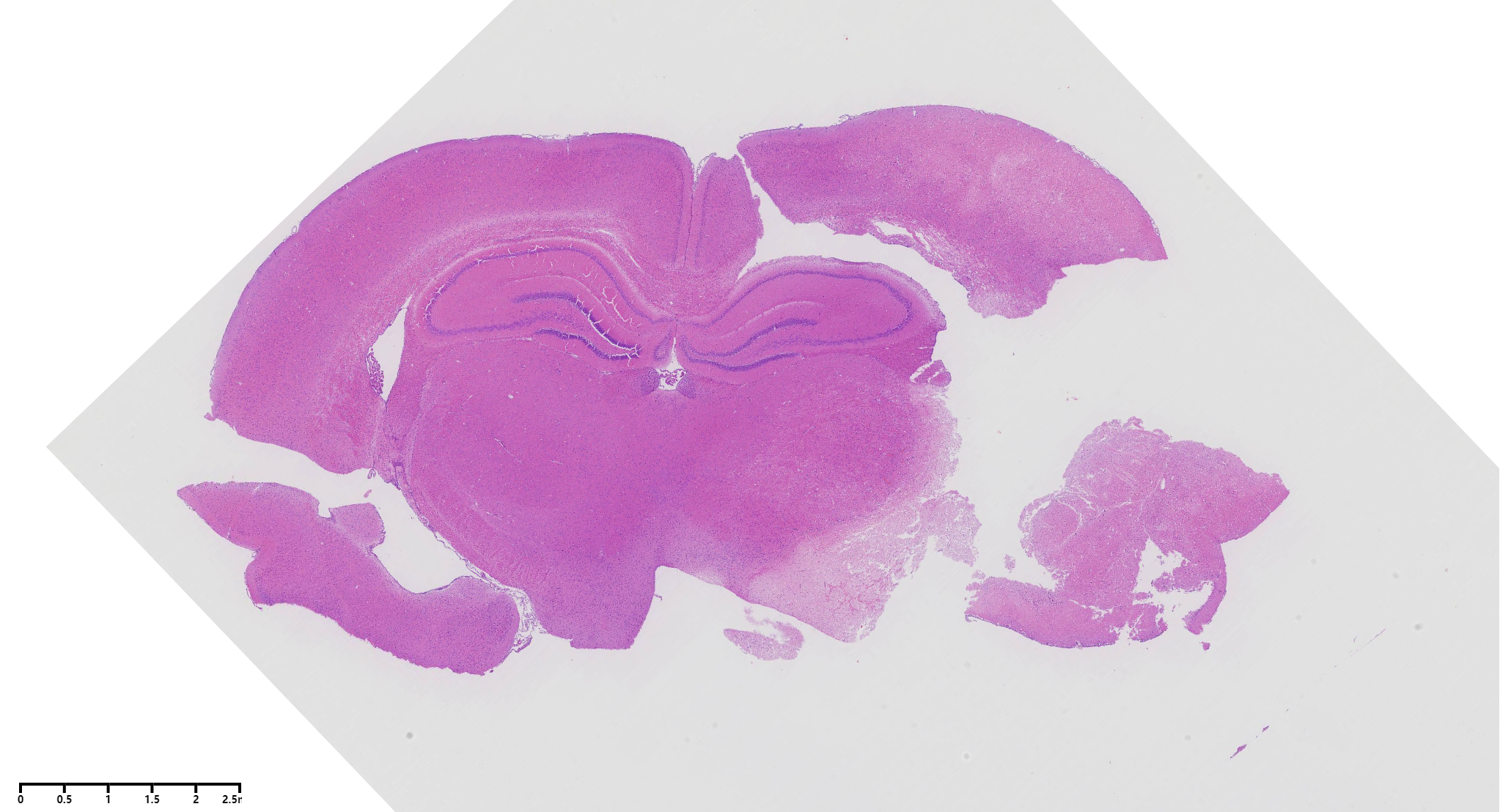

Supplement: Supplementary file 1 [file brainsci-14-01175-s001.zip › Figure 10.tif]

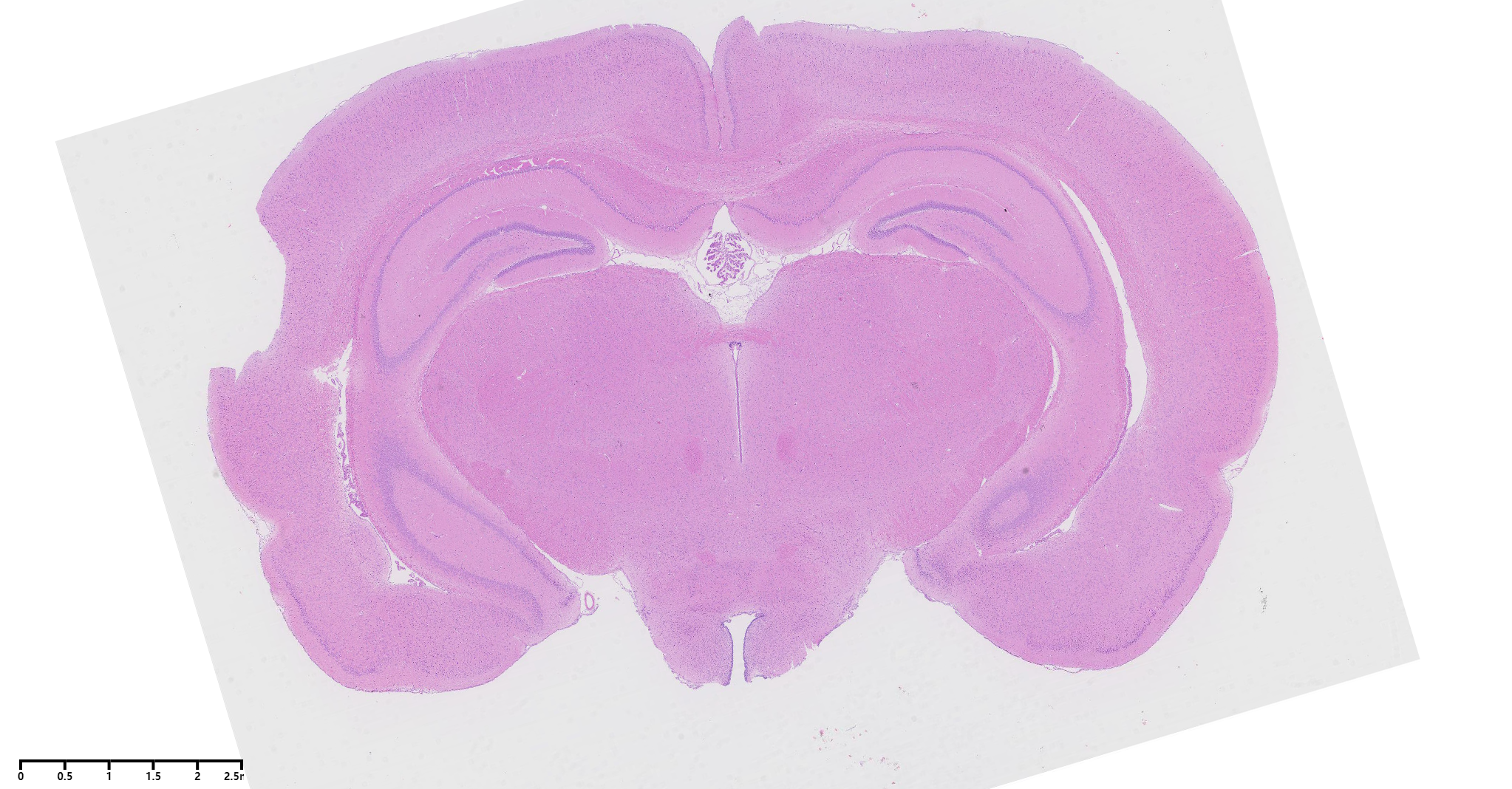

Supplement: Supplementary file 1 [file brainsci-14-01175-s001.zip › Figure 2.tif]

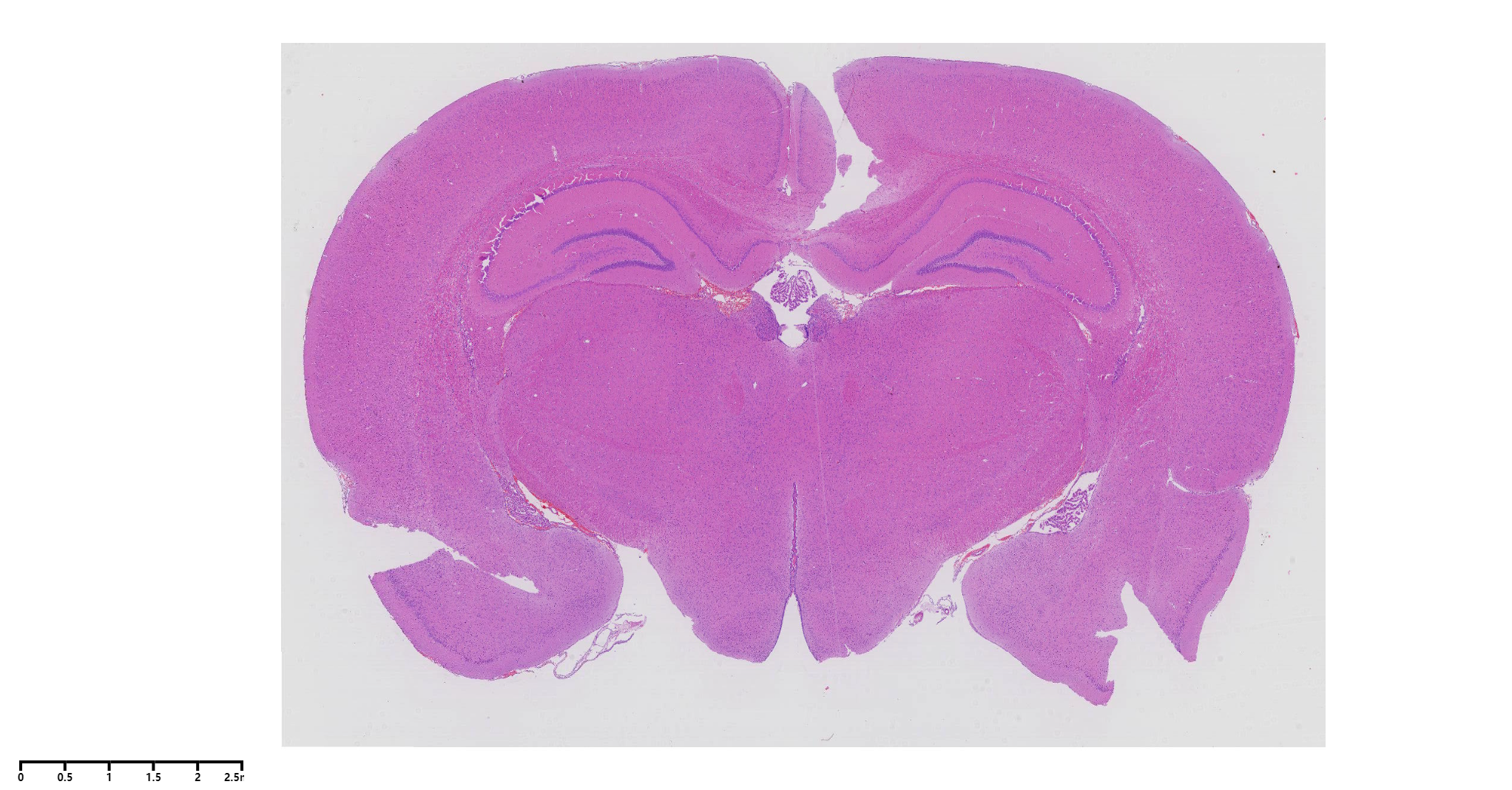

Supplement: Supplementary file 1 [file brainsci-14-01175-s001.zip › Figure 3.tif]

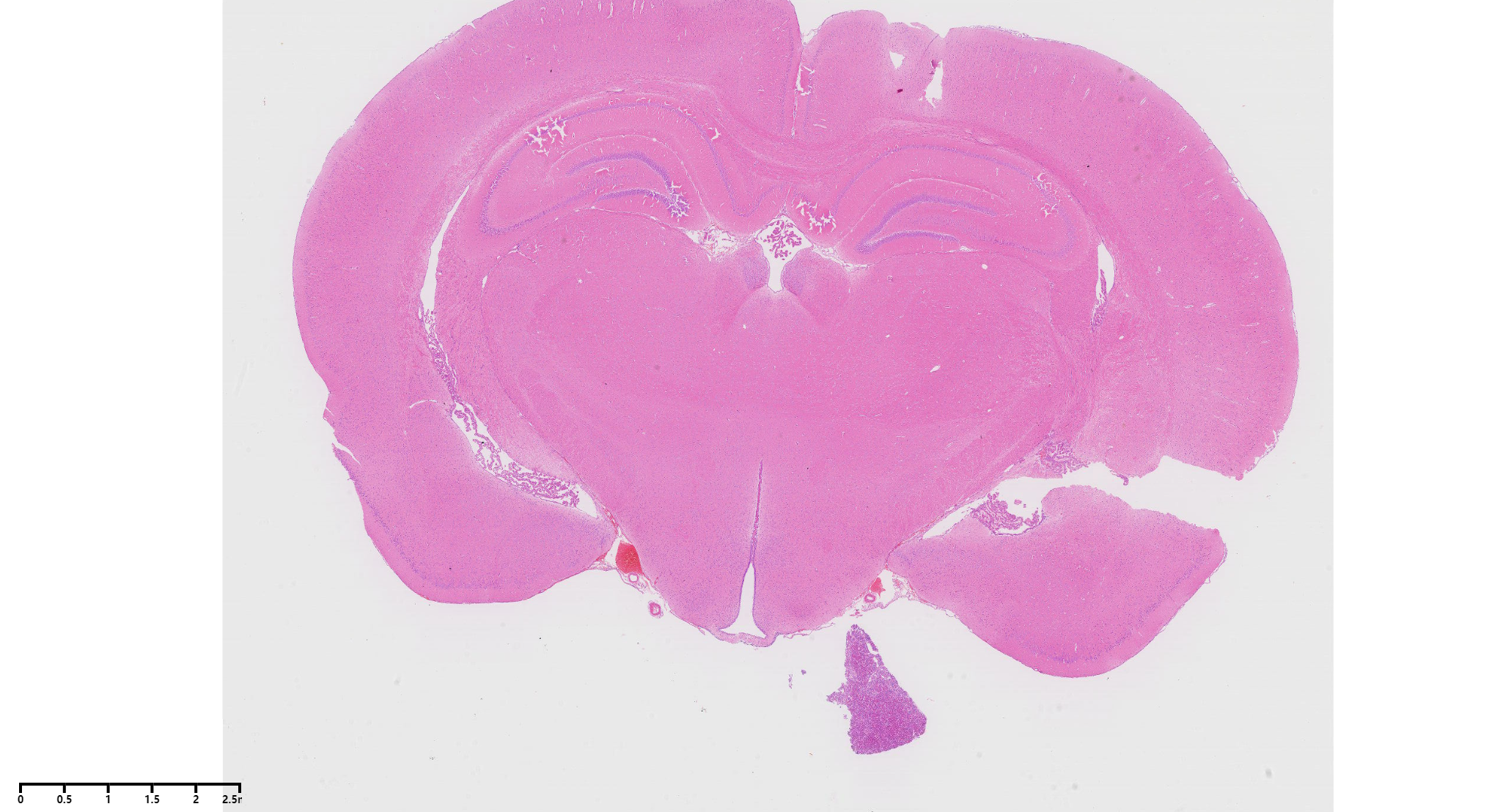

Supplement: Supplementary file 1 [file brainsci-14-01175-s001.zip › Figure 4.tif]

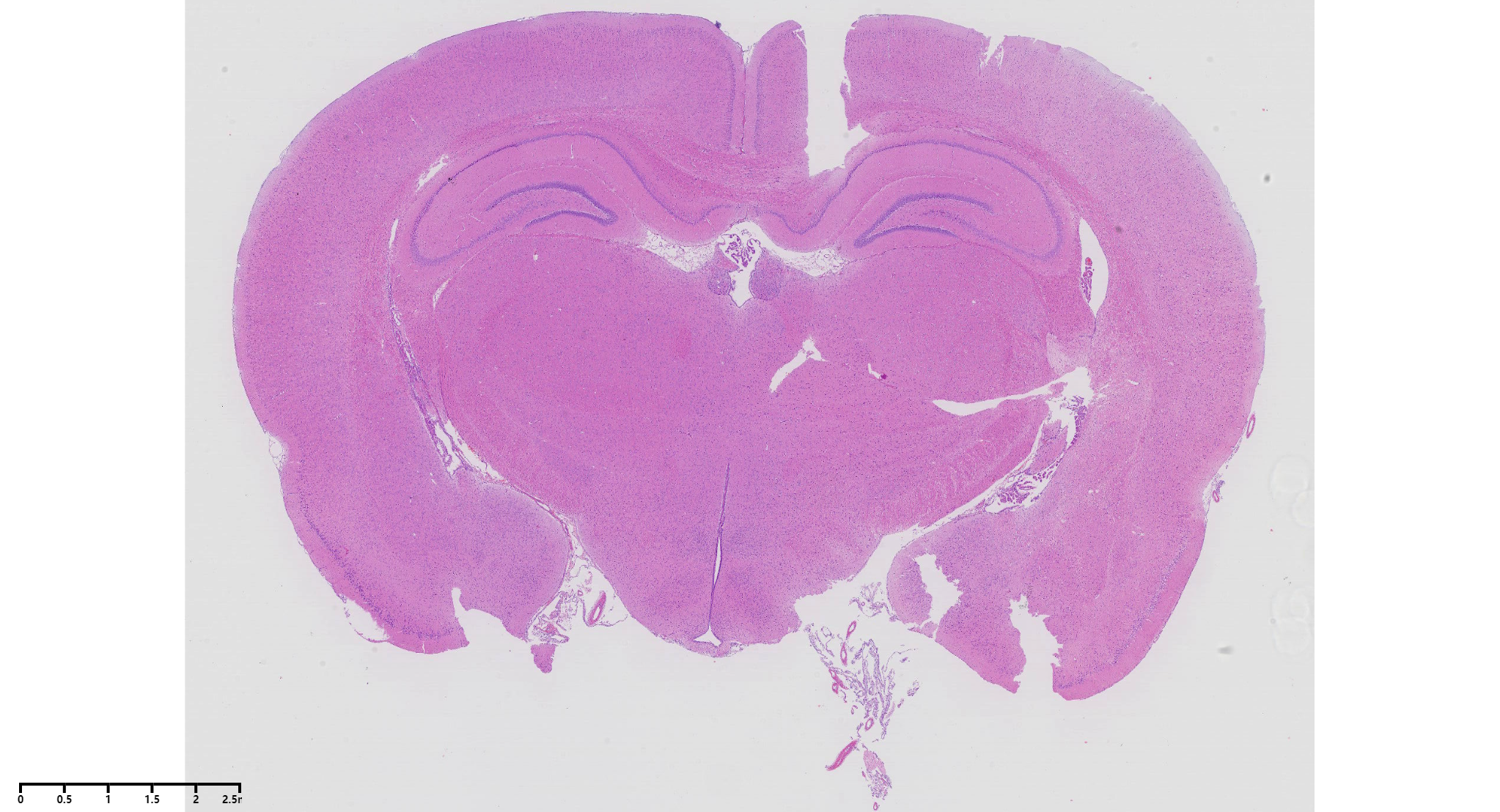

Supplement: Supplementary file 1 [file brainsci-14-01175-s001.zip › Figure 5.tif]

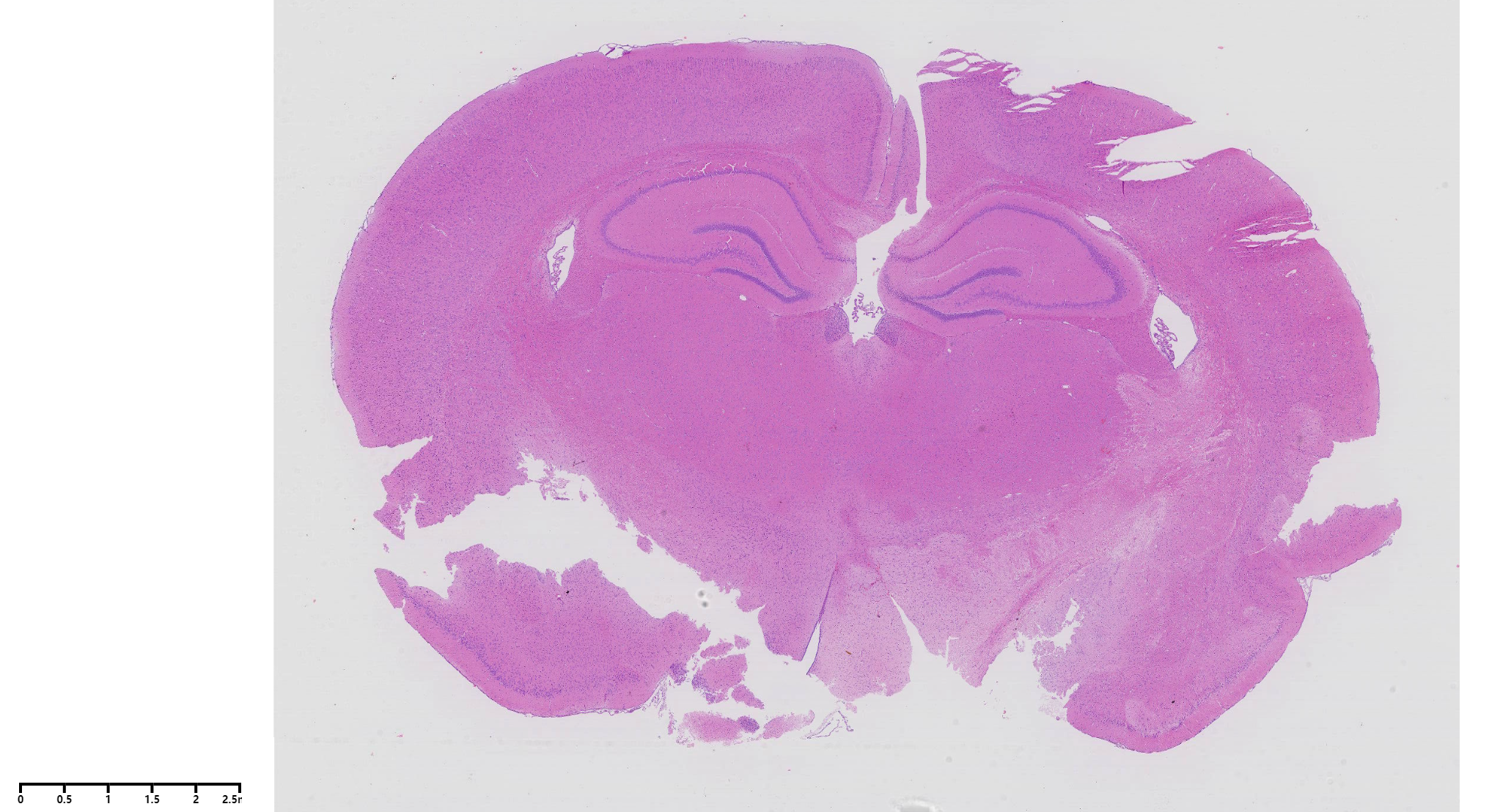

Supplement: Supplementary file 1 [file brainsci-14-01175-s001.zip › Figure 6.tif]

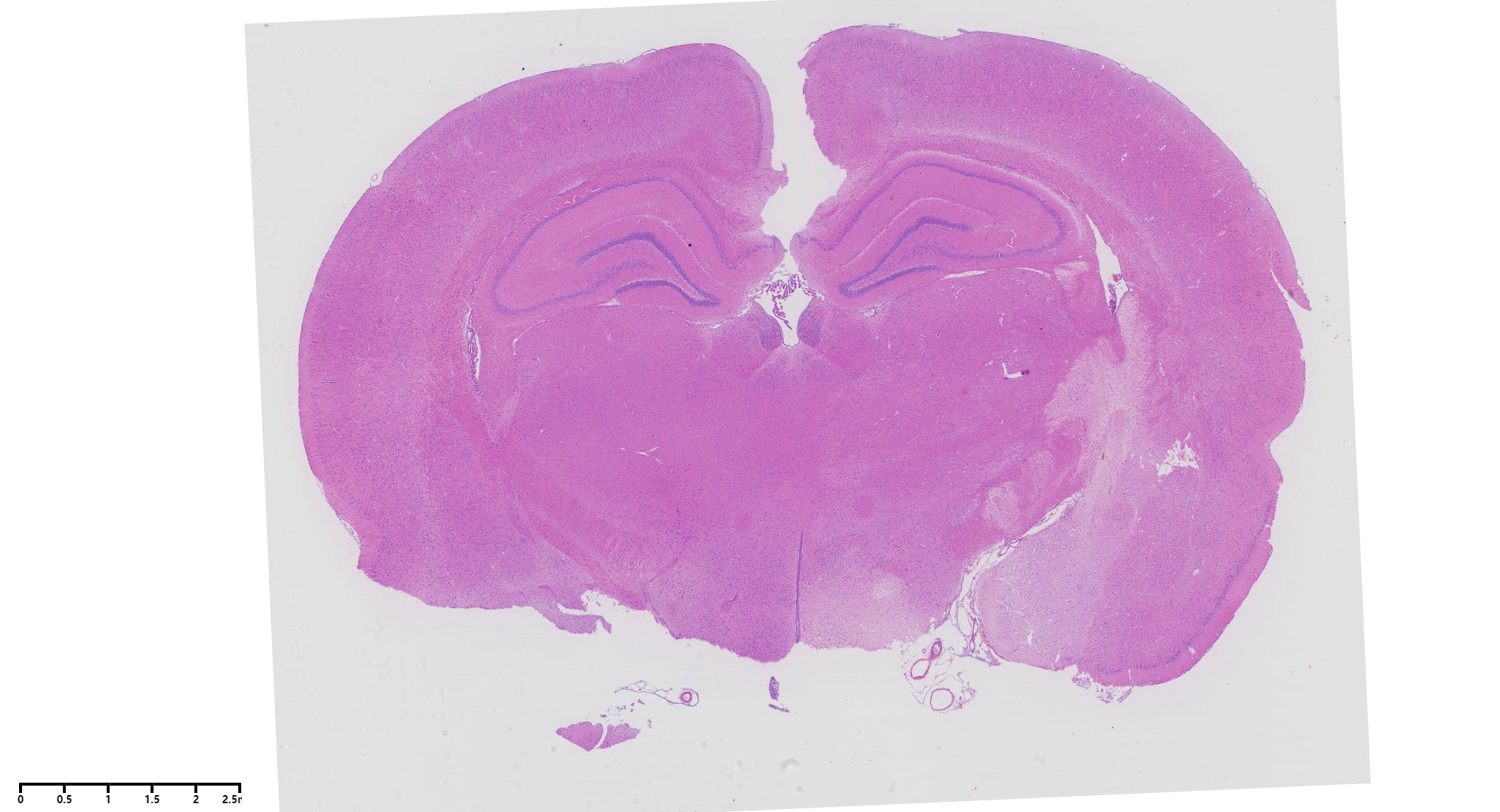

Supplement: Supplementary file 1 [file brainsci-14-01175-s001.zip › Figure 7.tif]

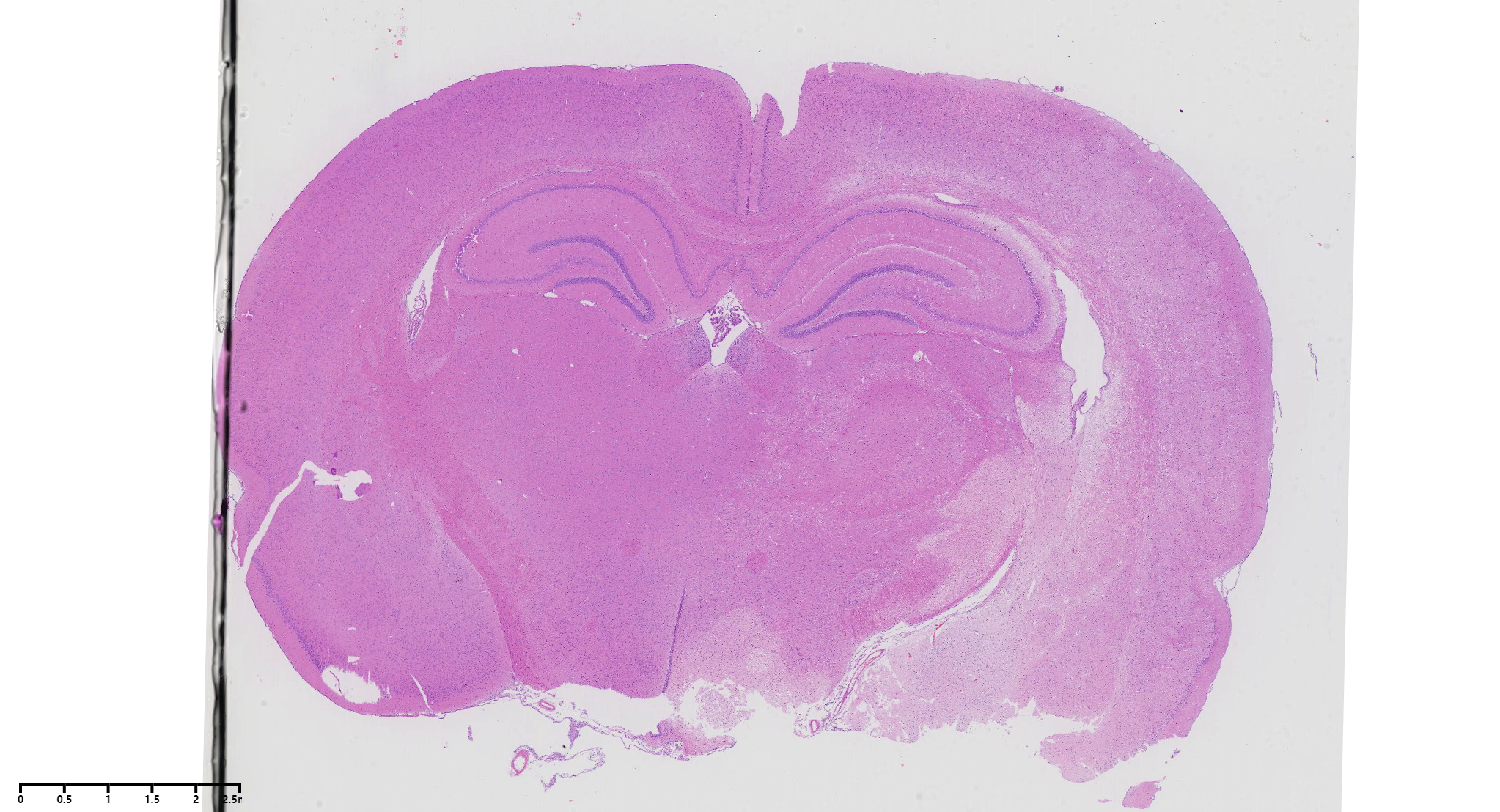

Supplement: Supplementary file 1 [file brainsci-14-01175-s001.zip › Figure 8.tif]

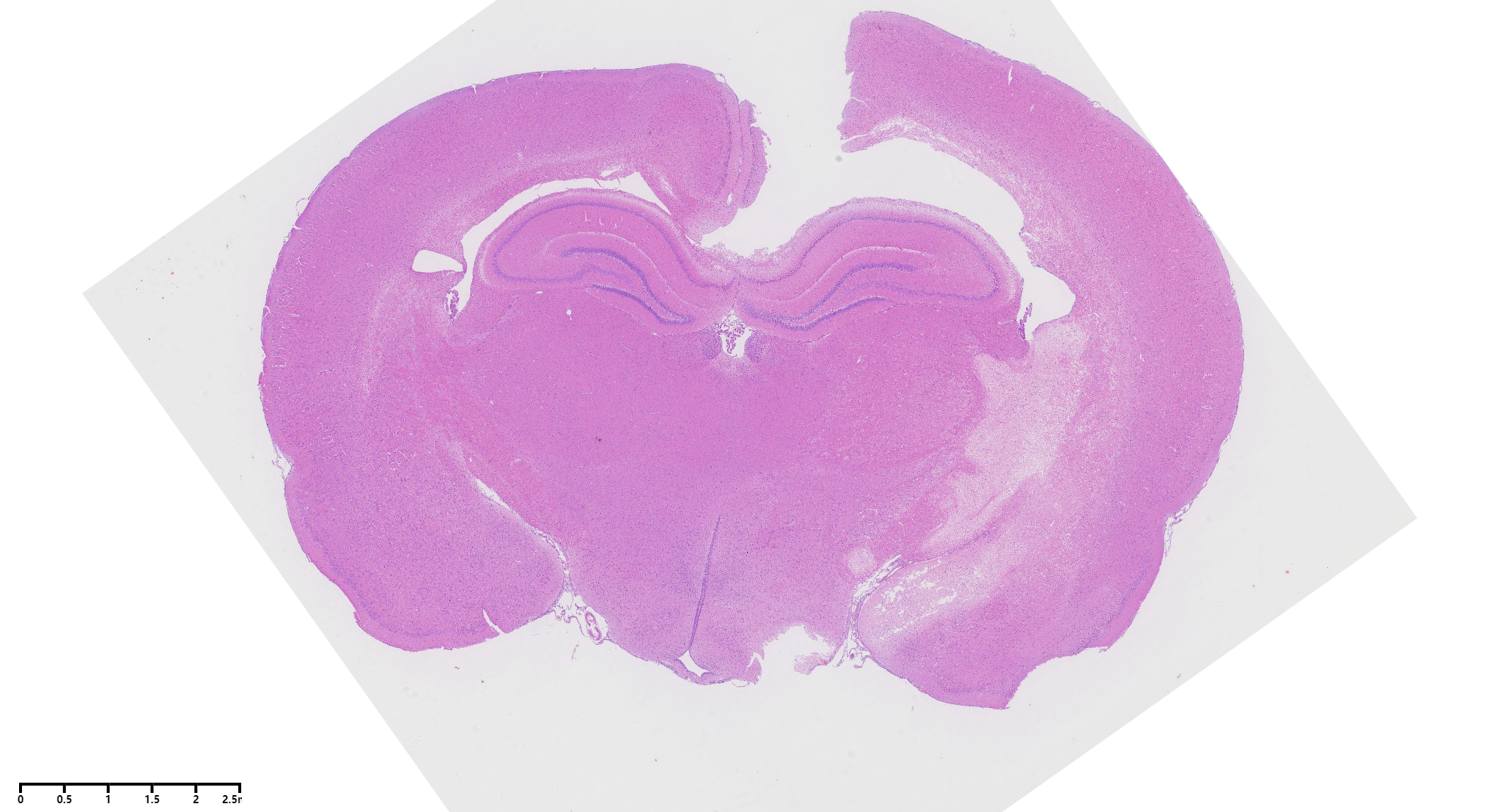

Supplement: Supplementary file 1 [file brainsci-14-01175-s001.zip › Figure 9.tif]
